# Structural basis for biomolecular recognition in overlapping binding sites in a diiron enzyme system

**DOI:** 10.1038/ncomms6009

**Published:** 2014-09-24

**Authors:** Justin F. Acheson, Lucas J. Bailey, Nathaniel L. Elsen, Brian G. Fox

**Affiliations:** 1 Department of Biochemistry, University of Wisconsin, Biochemistry Addition, 433 Babcock Drive, Madison, Wisconsin 53706, USA

## Abstract

Productive biomolecular recognition requires exquisite control of affinity and specificity. Accordingly, nature has devised many strategies to achieve proper binding interactions. Bacterial multicomponent monooxygenases provide a fascinating example, where a diiron hydroxylase must reversibly interact with both ferredoxin and catalytic effector in order to achieve electron transfer and O_2_ activation during catalysis. Because these two accessory proteins have distinct structures, and because the hydroxylase-effector complex covers the entire surface closest to the hydroxylase diiron centre, how ferredoxin binds to the hydroxylase has been unclear. Here we present high-resolution structures of toluene 4-monooxygenase hydroxylase complexed with its electron transfer ferredoxin and compare them with the hydroxylase-effector structure. These structures reveal that ferredoxin or effector protein binding produce different arrangements of conserved residues and customized interfaces on the hydroxylase in order to achieve different aspects of catalysis.

## Abstract (web summary)

The ability of a protein to interact with multiple other proteins is an intriguing problem. Here, the authors use crystallography to show how a diiron hydroxylase achieves two distinct steps in the catalytic reaction, by using an overlapping binding site to recognize two different binding partners.

## Graphical abstract

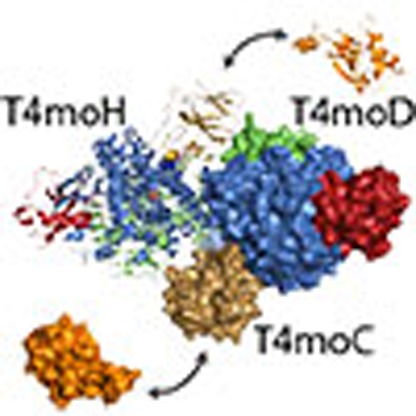

Protein–protein interactions are involved in nearly all parts of life from reproduction to central metabolism to function of the global carbon cycle. The molecular contacts made in binary pairs of proteins have been elucidated and many examples are known^1^. However, considerably less is known about how multiple proteins can use overlapping binding surfaces^2^. Diiron enzymes^3^ provide an example where multiple protein interactions are needed to achieve catalysis. These versatile complexes are involved in many critical biological processes including hydroxylation^4^,^5^ of the potent greenhouse gas methane^6^, aromatic compounds derived from lignin^7^ and pollutants^8^ and antibiotic precursors^9^, desaturation of fatty acids^10^,^11^ and synthesis of deoxyribonucleotides^12^,^13^.

Among the many different diiron enzyme complexes, the bacterial multicomponent monooxygenases (BMMs) have a unique requirement for both effector and redox proteins, as their absence hampers or eliminates catalysis^4^,^14^. Effector protein binding is associated with changing the redox potentials^15^ and spectroscopic signals from the mixed-valence and integer-spin states^16^,^17^,^18^,^19^,^20^ of the diiron centre, improving the coupling between electron transfer and hydroxylation^21^,^22^, influencing the timing of the O_2_ activation steps of the catalytic cycle^23^,^24^,^25^,^26^, and altering the regio-specificity of hydroxylation^22^,^27^. Toluene 4-monoxygenase (T4MO) is a member of these enzymes, and high-resolution structures of the hydroxylase T4moH (PDB: 3DHG), effector-hydroxylase T4moHD (PDB: 3DHH) and T4moHD-product (PDB: 3Q14) complexes are available^28^,^29^,^30^,^31^. These structures reveal conformational changes driven by T4moD binding occur along the substrate access channel, within the active site, and at the diiron centre^28^.

In the BMMs, electrons are typically provided by an flavin adenine dinucleotide- and [2Fe-2S]-containing NAD(P)H reductase^32^, although direct reduction of a diiron enzyme by NADH has also been observed^33^. In some BMMs, an additional electron transfer ferredoxin is needed to reduce the diiron centre^32^. For example, in the T4MO complex, T4moC (12 kDa)^34^ is a Rieske-type ferredoxin that forms a specific complex with T4moH^35^. However, structures of T4moHD show that effector protein binding covers all T4moH surface residues within ~20 Å of the buried diiron centre in a tightly matched protein–protein interface. Therefore, how T4moH forms an effective complex with T4moC, a protein with a different structural topology than T4moD, has not yet been clarified.

In this communication, we report X-ray crystal structures of the T4MO ferredoxin-hydroxylase electron-transfer complex (T4moHC) and compare them to the effector-hydroxylase O_2_-activation complex. The T4moHC structures reveal that a combination of proximal shape complementarity, unique positioning of conserved residues and hydrogen bonds connecting the [2Fe-2S] and diiron centres, and rearrangement of a surface loop from T4moH ~30 Å away from the redox centres give rise to a high-specificity electron transfer complex. In contrast, the T4moHD complex uses a larger surface area and specific steric contacts to collapse the substrate access channel and place both diiron ligands and active water molecules into a unique configuration presumably poised for O_2_ activation. In combination, these structures provide unique new insight into the ability of protein–protein interactions to optimize configurations of conserved residues and overlapping binding interfaces to achieve different aspects of catalysis.

## Results

### Structure determination

To gain insight into the protein interface used for electron transfer, we determined the structure of the hydroxylase-ferredoxin complex (T4moHC). Data collection and refinement statistics for two structures are presented in Table 1 (4P1C, P2_1_2_1_2_1_ space group, 2.4 Å; 4P1B, P3_2_21, 2.05 Å). In both diffraction data sets, the asymmetric unit contains the biological dimer, with the TmoA, TmoB, TmoE and TmoC polypeptides (ABEC protomer) arranged into an (ABEC)_2_ structure. All polypeptide chains, diiron centres and [2Fe-2S] centres have full occupancy. The two structures have minor differences in resolution, variant of T4moC used and whether acetate was present in the crystallization buffer. Further details of the structure determination can be found in Methods.

### Differences between 4P1C and 4P1B

T4moC used to obtain the lower resolution 4P1C structure (P2_1_2_1_2_1_ space group, 2.4 Å resolution) contains three mutations and acetate was not present in the crystallization buffers. The combination of Cys84Ala and Cys85Ala previously yielded a 1.4 Å structure of T4moC^36^; the third mutation, Cys7Ser, was created as part of this work to remove the remaining surface cysteine in order to eliminate possibilities for intermolecular disulfide bond formation. 4P1C contains no mutations in the biologically relevant HC interface. In the 4P1C active site, a polyethylene glycol (PEG) molecule has been modelled as a weakly coordinating μ-alkoxo ligand extending from the diiron centre into the substrate-binding cavity. Similar partially complete electron density assigned to exogenous ligands has been observed in other diiron hydroxylase structures^37^.

T4moC used to obtain the higher resolution 4P1B structure (P3_2_21, 2.05 Å resolution) contained an additional Glu16Cys mutation to potentially allow covalent crosslinking to mutated variants of T4moH, however, crystals were obtained without the use of chemical cross-linkers. This mutation resulted in a loss of a hydrogen bond between T4moC and TmoE, however, the position of binding of T4moC was nearly indistinguishable to that of 4P1C. In the 4P1B complex, acetate was included in the crystallization solutions, and yielded an acetate bound to the diiron centre as is often seen in other diiron enzymes and BMMs^38^. Root mean squared deviation (RMSD) comparisons of subunits in the two T4moHC structures are shown in Supplementary Table 1.

### Binding interface

**Figure 1:**
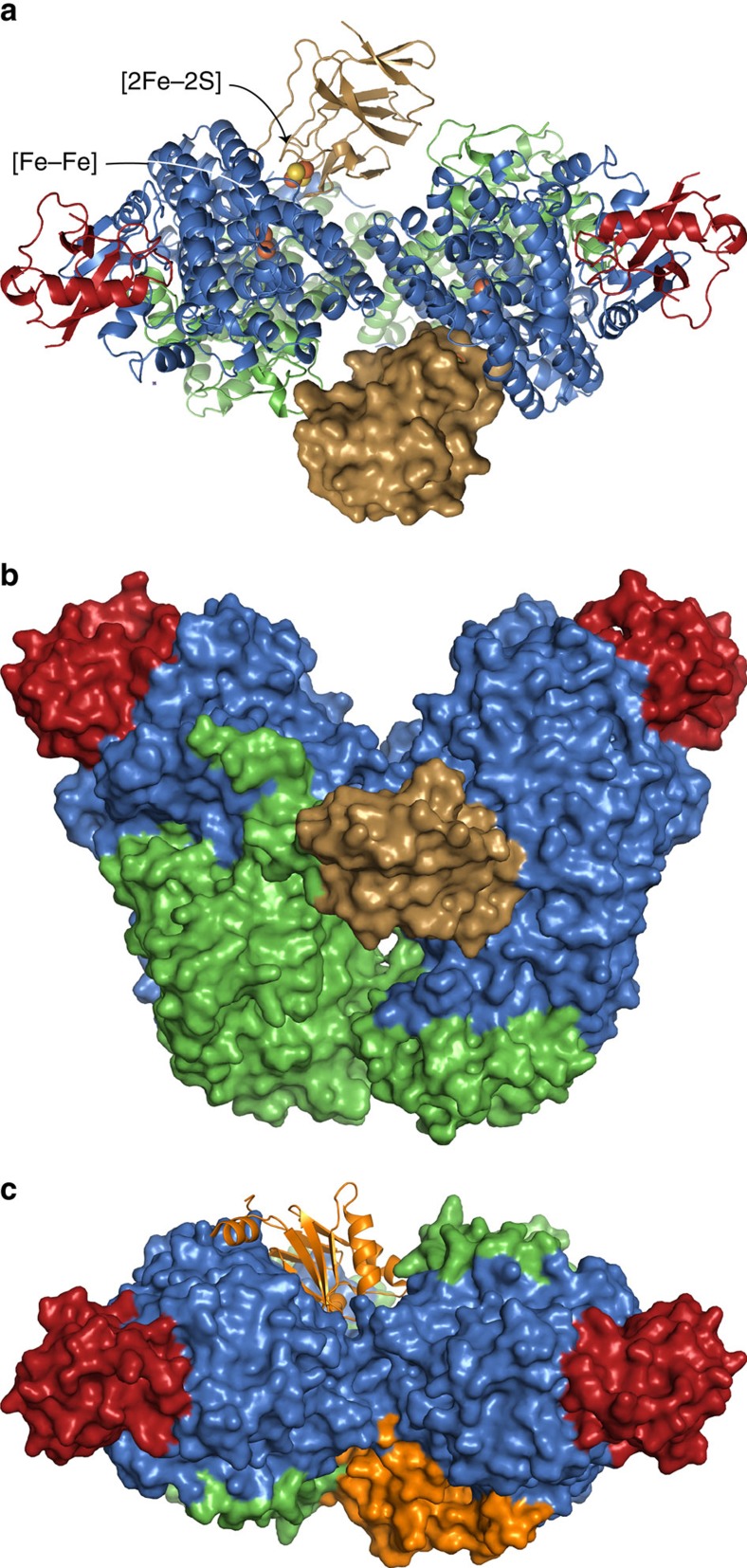
Structure of T4moHC complex. The T4moHC complex consists of two copies of the TmoA (blue), TmoB (red), TmoE (green) and Rieske-type ferredoxin T4moC (gold) polypeptides. (**a**,**b**) Two views given by 90° rotation about the *x* axis. The positions of the [2Fe-2S] cluster in TmoC, and the position of the diiron centre [Fe-Fe] in TmoA are shown as spheres in **a** to illustrate their relative positions in the complex. (**c**) T4moD (orange) is shown bound to the same surface of TmoA as T4moC.

**Figure 2:**
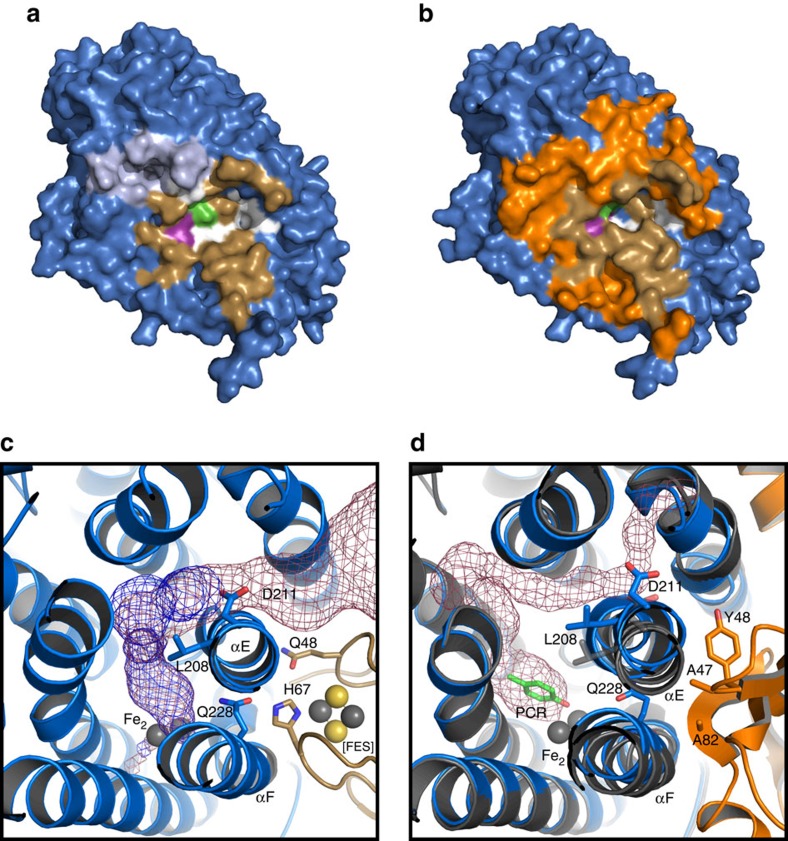
Surfaces and tunnels of TmoA. (**a**) Surface of TmoA (blue) alone, showing residues involved in T4moC binding (gold), entrance to the substrate channel (light blue), residues Asn202 (green) and Gln228 (purple), and six residues unique to the T4moHC interface (white). (**b**) Surface of TmoA from the T4moHD complex, showing 53 residues within 5 Å of T4moD that are unique to the T4moHD complex (orange), 17 residues in common with the T4moHC complex (gold), and six residues unique to the T4moHC complex (white). (**c**) Mesh mapping (raspberry and blue) of the open tunnel from solvent to the active site in TmoA from the T4moHC complex. (**d**) Mesh mapping of the closed tunnel in the T4moHD complex caused by repositioning of TmoA residues Leu208 and Asp211 relative to their positions in T4moHC (dark grey). These shifts are produced by steric contacts with T4moD residues Ala47 and Tyr48, causing complete collapse of the entrance to the substrate channel at the fork position.

T4moC binds in the canyon formed at the dimer interface of T4moH, making extensive contacts with TmoA (Fig. 1a and Supplementary Fig. 1a). Furthermore, T4moC bridges to TmoE of the opposite protomer of T4moH to recognize the quaternary structure of the hydroxylase. The T4moHC complex utilizes a smaller protein–protein interface with an area of ~1150 Å^2^ and the interface is dominated by electrostatic interactions (Fig. 2a and Supplementary Fig. 1a). There are 23 residues from TmoA within 5 Å of T4moC; 12 are also within 15 Å of the diiron centre of TmoA. Six deeply recessed residues, including diiron centre ligand Glu231, are uniquely involved in the T4moHC complex. Consequently, the T4moHC interface includes all TmoA residues that provide closest approach from the surface to the diiron centre.

For comparison, the T4moHD interface (~2,200 Å^2^) is ~2 × larger than the T4moHC interface (Fig. 2b). In T4moHD, 53 residues from TmoA are within 5 Å of T4moD, including both polar and non-polar residues (Fig. 2b and Supplementary Fig. 1b). Seventeen of these residues are also found in the T4moHC interface; however, a water-filled cavity overlays the six most deeply recessed residues from TmoA in the T4moHD complex.

### Inhibition by T4moD

T4moD activates toluene hydroxylation at low concentrations and inhibits at high concentrations^22^. Kinetic studies show that T4moD acts as a competitive inhibitor of T4moC by increasing the apparent *K*_M_ (~3 μM increased to 16 μM) while yielding the same *k*_cat_ (~3 s^−1^ per T4moH) at saturating T4moC (Supplementary Table 2 and Supplementary Fig. 2a). The *K*_I_ for T4moD was also increased from 45±7 to 240±50 μM when [T4moC] was increased from 12 to 42 μM (Supplementary Table 2 and Supplementary Fig. 2b). These steady-state kinetic behaviours are consistent with the overlap in binding sites established by the structures of T4moHC and T4moHD (Fig. 1).

### Substrate channels

As first shown in toluene/*o-*xylene monooxygenase^39^, T4moH has a hydrophobic tunnel with a forked entrance that allows access to the active site^28^. T4moC binding leaves this tunnel open (Fig. 2c) and in a nearly identical configuration to T4moH. With the tunnel open, substrates may enter and product may leave through the forked position controlled by residues L208 and D211 (Fig. 2c). T4moD binding shifts helix E (Fig. 2d), which moves these gating residues and collapses the active site access at the fork position.

### Distal contribution to T4moC binding

**Figure 3:**
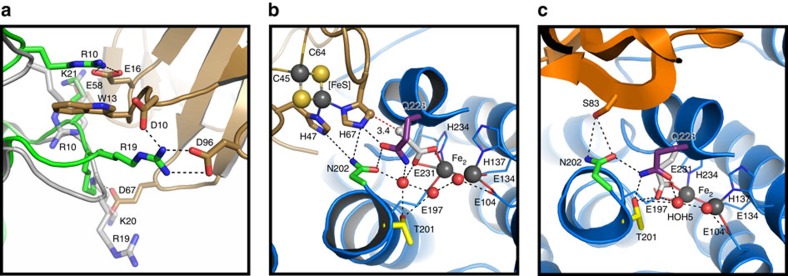
Effects of T4moC and T4moD binding to T4moH. (**a**) Binding site for T4moC Trp13 formed from TmoE residues 10–21 (green cartoon with sticks shown for Arg10 and Arg19 that provide stabilizing H-bonding interactions with T4moC Glu16, Asp10 and Asp96). Another view of this interface shows the positions of the TmoE loop residues in the T4moHD complex (PDB accession 3DHH) are shown in Supplementary Fig. 3. (**b**) Electron transfer interface in T4moHC including T4moC (gold) and T4moH diiron ligands (blue), E231 (white), Asn202 (green) and Gln228 (purple). The distance from the [2Fe-2S] to the diiron centre is ~12 Å, and the closest contact between T4moC His67 and TmoA Glu231 is indicated by a dashed red line (3.4 Å). (**c**) T4moD binding and hydrogen bonding rearrangement poises a catalytic water HOH519 for O_2_ activation.

The T4moHC structure revealed that T4moC bridges between (ABE) protomers in T4moH. Loop residues 10–21 (RTWSHLAEMRKK) from the opposite TmoE make electrostatic contacts with T4moC and also form a hydrophobic loop surrounding T4moC Trp13 (Fig. 3a). Thus, molecular recognition of the ferredoxin is enhanced through interaction with the hydroxylase over 30 Å distal from the primary interface. The TmoE loop residues adopt different configurations in both the uncomplexed T4moH and the T4moHD complex structures (Supplementary Fig. 4). In the T4moH crystals (PBD 3DHG), the loop makes several contacts with an adjacent dimer in the crystal lattice. In solution, however, the TmoE loop would be solvent exposed and likely disordered or able to sample multiple conformations^40^. When T4moD binds (PDB 3DHH), this loop adopts a third conformation as a result of minor binding interactions with the effector (Fig. 3a and Supplementary Fig. 4).

### Electron transfer

The distance between redox partners is an important factor in efficient electron transfer. In the T4moHC complex, the distance between the [2Fe-2S] cofactor and diiron centre is ~12 Å (Fig. 3b and Supplementary Fig. 5), a typical distance compared with other biological electron transfer complexes^41^,^42^. HARLEM predicts a shortest gap 3.4 Å electron transfer (Fig. 3b) between His67 of T4moC and Glu231 of TmoA, which are ligands to the [2Fe-2S] and diiron centres, respectively^43^. A hydrogen-bonding network, which is generated in the protein interface, connects residues His67 of T4moC to Gln228 of TmoA and then to Fe2 ligand Glu231.

### Outer sphere active site residues

A triad of non-ligand TmoA residues, Thr201, Asn202 and Gln228, are important in catalysis^28^,^29^,^44^. The involvement of these residues in positioning active site water is consistent for both T4moHC and T4moHD (Fig. 3b,c), but with several differences revealed by the structures. Relative to their positions in T4moHD (Fig. 3c), Thr201 is shifted by ~2 Å inside the T4moHC active site (Fig. 3b), whereas Asn202 and Gln228 are displaced by ~3 Å and ~2 Å, respectively, in order to accommodate His67 of T4moC, a ligand to the [2Fe-2S] cofactor. Along with the carbonyl oxygen of Fe2 ligand Glu197, this triad holds HOH506 ~4 Å from Fe2 (Fig. 3b). Importantly, Glu231 in T4moHC has bidentate coordination to Fe2, thus cannot provide hydrogen bonds to either Thr201 or an active site water molecule, which are both observed in the T4moHD complex.

**Figure 4:**
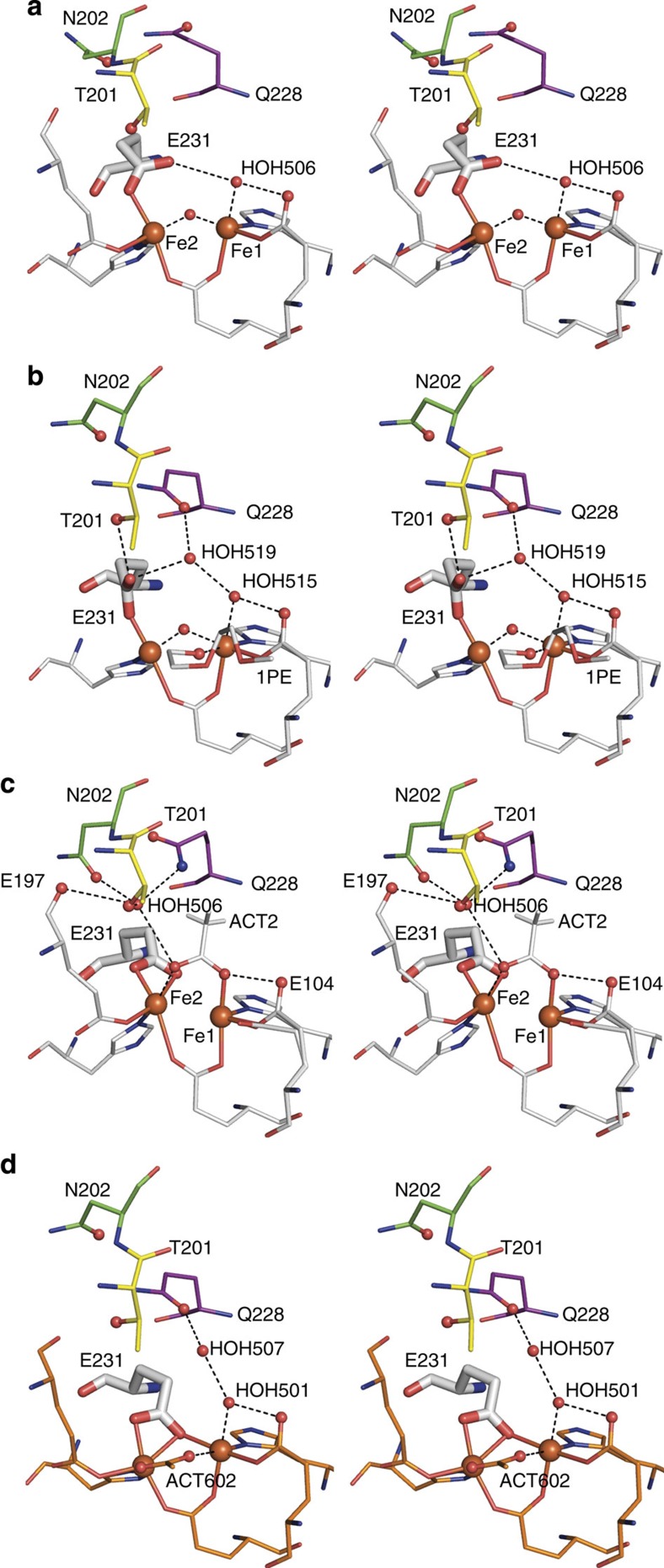
Stereo diiron centre configurations in different complexes and redox states of T4moH. Positions of key residues Thr201 (yellow), Asn202 (green), Gln228 (purple), Glu231 (white) shown as sticks. Unique active site water molecules are identified by their PDB ID numbers. (**a**) Resting T4moH (PDB 3DHG) with diferric centre and iron atoms Fe1 and Fe2 identified. (**b**) Resting T4moHD (PDB 3DHH) with diferric centre and polyethylene glycol (1PE) bound to Fe1 and projecting into the substrate cavity. (**c**) Resting T4moHC (PDB 4P1B) with acetate (ACT2) bound in a bridging position between Fe2 and Fe1. (**d**) Reduced T4moHD (PDB 3DHI) with acetate (ACT605) bound in a bridging position that projects into the substrate cavity.

No waters are present in T4moHC at a position comparable to where HOH519 and HOH507 are found in the T4moHD and reduced T4moHD complexes, respectively (Figs 3b,c and 4)^28^,^29^. We have hypothesized that the uniquely positioned water in T4moHD is a potential candidate for proton donation during catalysis, perhaps to facilitate cleavage of the O–O bond in a peroxo-diferric complex^30^, or to maintain charge balance as the p*K*_a_ of metal-bound water/hydroxide responds to changes in redox state of the diiron centre.

### Active site coordination

The T4moHC complex has a unique active site geometry compared with other T4moH structures (Fig. 4 and Supplementary Fig. 6). With the exceptions of Glu104 and Glu231, other diiron centre ligands have nearly identical geometries to those observed previously. In the higher-resolution 4P1B structure, Glu104 is present in two equal-occupancy configurations with the carboxyl groups rotated ~90° relative to each other. Acetate is also observed as a bridging ligand to the diiron centre. Glu231 was modelled in a single, bidentate configuration that is similar to the geometry of the homologous Glu243 observed in crystals of mixed valence methane monooxygenase^45^. This configuration is distinct from the monodentate geometry observed in resting T4moH (PDB 3DHG, Fig. 4a) other diferric BMMs^38^,^39^,^46^ and the bidentate carboxylate-bridging geometry in reduced T4moHD (PDB 3DHI, Fig. 4d) and other BMMs^45^.

## Discussion

The T4moHC complex reveals, to the best of our knowledge, the first structural account of how overlapped binding sites can be shared in a multicomponent enzyme. The combination of shape complementarity, unique configurations of three conserved residues and their hydrogen bonds in the interfaces used for either electron transfer or O_2_ activation, and a distal, binding-induced rearrangement are used to achieve both high specificity and efficiency of the enzyme reaction with toluene.

Shape complementarity in the deepest portion of the binding interface allows the ferredoxin [2Fe-2S] centre to achieve the closest possible through-space approach to the hydroxylase diiron active site, whereas the unique 4-,5-coordinate geometry produced by T4moC binding (Fig. 4c) likely represents a new diiron centre configuration involved in electron transfer. In the outer portion of the overlapping binding surface, rearrangements of alpha helices on the hydroxylase place a triad of conserved hydroxylase residues (Thr201, Asn202, Gln228) into a hydrogen-bonded electron transfer interface that connects the [2Fe-2S] and diiron centres and entrains a water molecule that may help to maintain charge balance during electron transfer. This triad has a different arrangement in the hydroxylase-effector complex^28^, which plausibly serves to position diiron ligand Glu231 and active site water for O_2_ activation, proton transfer and substrate hydroxylation steps of the catalytic cycle^8^,^14^,^22^,^26^.

Figure 4 shows that Glu231 has a different coordination geometry in T4moH (Fig. 4a, resting diferric state), T4moHD (Fig. 4c, diferric state) and reduced T4moHD (Fig. 4d, diferrous state produced in the presence of excess sodium dithionite). Thus, changes in T4moH coordination are produced both by protein binding and by changes in redox state of the diiron centre. The bidentate geometry of Glu231 in T4moHC is most similar to the coordination observed for homologous Glu243 in methane monooxygenase crystals treated with redox mediators so as to stabilize a 1e^–^ reduced, mixed valence state^45^. In the absence of spectroscopic evidence obtained on T4moHC crystals, assignment of the redox state of a diiron centre in a crystal is an inferential process. Because ferredoxins are known to be reduced by synchrotron radiation^47^, it is possible that T4moHC contains either a diferric or a mixed valence diiron centre, but less likely a diferrous centre because the bidentate carboxylate-bridging geometry is not present.

Acetate has been frequently used as an additive in the crystallization of diiron enzymes^28^,^38^. In 4P1B, acetate formed a bridge between Fe1 and Fe2, with the methyl group uniquely pointing towards TmoA Gln228 (Fig. 4c). In other diiron enzyme structures, Fe-bound acetate projects away from the diiron centre into the substrate binding cavity, a ~90° reorientation relative to that observed in 4P1B. Also in 4P1B, Glu104 showed two conformations with occupancies of 61/39 in chain A and 53/47 in chain D. The majority conformation was hydrogen bonded to the bound acetate, whereas the other conformation had the carboxylate rotated 90° towards the substrate-binding cavity with respect to previous structures. In the rotated conformation, the carboxylate group was hydrogen bonded to HOH966 that also weakly interacted with Fe1 (2.7 Å) and Fe2 (3.3 Å). These new configurations further demonstrate the influence of ferredoxin binding on the properties of the diiron centre.

Control of substrate access is presumed necessary to promote coupling of diiron centre reduction with substrate hydroxylation^31^,^39^. Binding of T4moD shifts helix E (Fig. 2d), effectively trapping substrate in the active site and deterring uncoupled electron consumption. Release of product from the diferric centre may be facilitated by reduction and the open egress in T4moHC^31^. Consequently, the T4moHC complex enables both access and egress to the active site and also electron transfer, whereas the T4moHD complex restricts access to the active site but poises the complex for catalysis by repositioning the diiron centre ligands and active site waters^28^,^31^. The requirement for separate effector and redox partners to control active site access, provide electron transfer and rearrange the position of conserved active residues for catalysis distinguishes the BMMs from P450s, which use only a ferredoxin to achieve both effector and redox functions without major conformational changes^48^.

Previous studies with other Rieske-type ferredoxins showed that TbuB, the Rieske-type ferredoxin from *Ralstonia pickettii* toluene monooxygenase (46% sequence identity, Swissmodel^49^, QMEAN score-0.2), was able to substitute as the proximal electron donor for T4moC in catalysis, whereas BphF from *Burkeholderia cepacia* LB400 biphenyl dioxygenase (26% sequence identity, 0.8 Å rms deviation, PDB 1FQT) could not^35^,^50^. The present structure provides insight into these prior results. In a homology model with T4moC, TbuB has identical residues in nearly every interface position, including T4moC Trp13 that interacts with the adjacent protomer. Furthermore, substitution of T4moC Val14 by TbuB Glu14 may allow formation of an additional salt bridge with TmoE Lys20 on the adjacent protomer. With this similarity, it is likely the interactions with the flexible loop from TmoE observed in T4moHC will also occur with TbuB. Minor differences include the substitution of T4moC Asp83 with TbuB Gly82, resulting in the loss of an electrostatic interaction, and two fewer residues at the C-terminus, leading to a loss of favourable packing interactions with TmoA Pro50 and TmoA Gln336. These changes may account for the modest increase in apparent *K*_M_ for TbuB. In contrast, BphF is missing a loop-organizing hydrophobic residue analogous to T4moC Trp13, and so may not induce the stabilizing disorder-to-order transition. BphF also has a loop containing three Pro residues, which would unfavourably protrude into the electron transfer interface at TmoA Tyr51. The loss of key interactions that help to anchor the ferredoxin and likelihood of steric clashes in the interface provides a structural basis for inability of BphF to act as an effective reductant for T4moH.

The functional imperative to bind structurally distinct proteins to the same docking surface presents an interesting dilemma in biomolecular specificity. In methane monooxygenase, effector protein binding advances the catalytic cycle by enhancing the rate of formation of several increasingly oxidized intermediates (diferrous-O_2_ adduct (P*)^26^; diferric peroxo (P)^51^; and diferryl (Q)^51^) formed after combination of the diferrous centre with O_2_. However, reduction of the diferric hydroxylase to the diferrous state, which is required to initiate the catalytic cycle, also apparently requires disassociation of the tightly bound effector (*K*_D_=70 nM (ref. 24) so that the ferredoxin (reductase) may bind. In T4MO, the overlapped binding sites for effector and ferredoxin with hydroxylase may be a key element in redox gating of the catalytic cycle, with formation of diferrous centre controlled by competitive formation of either a non-productive diferric T4moHD complex or a complex with 1e^−^ reduced T4moC at the same surface of T4moH and involving the same conserved residues. Preferential reduction to the diferrous state may arise from an ~10^4^-fold weaker affinity of the effector as the hydroxylase is reduced^52^, which would otherwise block reduction at the overlapping sites, and by an increase in the redox potential of the diiron centre correlated with rearrangements of the diiron centre ligands. Subsequent combination of the diferrous centre with O_2_ would initiate the oxidative steps of catalysis, and an increase in the affinity of effector would productively drive the catalytic cycle forward^24^. Presence of a tightly bound effector during this stage of catalysis would also help to prevent adventitious reduction of the activated intermediates^21^,^53^.

Here we have shown how binding of either effector or ferredoxin to an overlapping surface on the diiron hydroxylase can be achieved. Although effector protein binds tightly to a large surface area and induces a conformational change extending into the hydroxylase active site^28^, ferredoxin binding combines rearrangement of a loop ~30 Å distal with a tightly matched, hydrogen bonded contact surface that places the [2Fe-2S] and diiron centres in their closest possible approach for electron transfer. The differences provided by a combination of intricate shape matching, alternative conformations of a conserved triad of residues and flexible accommodation provides an exquisite example of how specificity can be achieved in biomolecular catalysis.

## Methods

### Cloning

All mutagenesis experiments were carried out by Quickchange (Stratagene) and sequence-verified at the University of Wisconsin Biotechnology Center. Vector pVP58kABE (T4moH) was modified to contain stop codons at codon 492 in the *tmoA* gene and codon 307 in *tmoE* gene in order to eliminate C-terminal sequences that were disordered in all prior crystal structures. Vector pET28T4moCD, encoding C84A/C85A mutations in T4moC and also T4moD, was modified to also encode an E16C mutation in T4moC. Both the pET28T4moCD and pET28T4moCD E16C plasmids were modified to encode a C7S mutation to remove the only remaining surface cysteine from T4moC.

### Protein expression

pVP58kABE was transformed into *Escherichia coli* BL21 and the T4moCD vectors were transformed into *E. coli* BL21(DE3). The transformation reaction was plated onto Luria-Bertani agar plates containing 50 μg ml^−1^ of kanamycin and grown overnight at 37 °C. A starting inoculum for the stirred vessel fermentation was prepared by placing a single colony into 2 ml of non-inducing medium containing (NH_4_)_2_SO_4_, KH_2_PO_4_, Na_2_HPO_4_, aspartate, amino acids, and glucose and 50 μg ml^−1^ of kanamycin^54^. The 2-ml culture was incubated at 37 °C with shaking at 250 r.p.m. until it reached an OD_600_ between 0.5 and 1. Next, two 2 l flasks were prepared containing 500 ml of the same medium and 50 μg ml^−1^ kanamycin was inoculated with ~500 μl of the 2 ml culture and incubated overnight (~12 to 14 h) at 25 °C with shaking at 250 r.p.m. When the overnight cultures had reached an OD_600_ ~1, they we added to 9 l of medium in a Bioflo 110 fermenter (New Brunswick Scientific) and grown at 37 °C with agitation at 300 r.p.m. The dissolved O_2_ content was allowed to decrease to 20% of air saturation and subsequently, the agitation was allowed to increase under feedback control to maintain the dissolved O_2_ content at 20% of air saturation. At an OD_600_ ~3, the temperature was decreased to 25 °C and 0.4 g of isopropyl-β-D-thiogalactoside (Fisher Scientific) dissolved in 10 ml of ddH_2_O, 20 g of Casamino acids (Fisher Scientific) and 36 g of lactose were added to the culture medium. After induction of protein expression, the culture growth was continued for 5 h, and the OD_600_ reached ~8. The cells were recovered by centrifugation at 4,200*g* for 25 min at 4 °C in a JS-4.2 rotor and an AvantiTM J-HC centrifuge (Beckman Coulter). The yield from this expression protocol was ~8 to 9 g of wet cell paste per litre of culture medium. The cell paste was stored at −80 °C.

### Purification of T4moH

T4moH cell paste was re-suspended in 25 mM MOPS, pH 6.9, containing 150 mM NaCl and 2% glycerol at a ratio of 1.5 ml per g of wet cell paste^31^. The cell suspension was sonicated on ice at high intensity for 8 min (15 s on; 30 s off). The supernatant from the sonicated cells was recovered by centrifugation at 39,200 *g* for 60 min at 4 °C. The supernatant was carefully decanted and diluted with two volumes of the above buffer and loaded onto a DEAE Sepharose column (45 mm diameter × 250 mm, GE Health Care) equilibrated in the above buffer and eluted in a 150–450 mM NaCl gradient in the same buffer at a linear flow rate of 40 cm h^−1^. Fractions were pooled based on both activity and purity as determined by SDS–polyacrylamide gel electrophoresis (SDS–PAGE. Pooled fractions were concentrated and applied to a Sephacryl S-300 (45 mm diameter × 1,000 mm, GE Health Care) column equilibrated in 25 mM MOPS, pH 7.5, 200 mM NaCl and 5% glycerol at a flow rate of 5 ml min^−1^. Fractions were pooled based on activity and purity, concentrated to ~800 μM active sites, and exchanged into 10 mM MOPS, pH 6.9, containing 200 mM NaCl. The purified protein was drop frozen in liquid N_2_ and stored at −80 °C.

### Purification of T4moC and T4moD

Cell paste containing a co-expression of T4moC and T4moD was re-suspended in 25 mM MOPS, pH 7.5, containing 2% glycerol but no NaCl at a ratio of 1.5 ml per g of wet cell paste^31^. The cell-free extract was prepared as described above for T4moH. The supernatant was carefully decanted and diluted with two volumes of the above buffer and loaded onto a DEAE Sepharose column (45 mm diameter × 250 mm, GE Health Care) equilibrated in the above buffer and eluted in a 0–450 mM NaCl gradient in the same buffer at a linear flow rate of 5 ml min^−1^. T4moD fractions were pooled based on activity and T4moC was pooled based on the brown colour of the [2Fe-2S] cofactor. Purity of both proteins was monitored by SDS–PAGE. Pooled fractions were individually concentrated and applied to a Sephacryl S-100 (45 mm diameter × 1,000 mm, GE Health Care) column equilibrated in the above buffer at a linear flow rate of 3.33 ml min^−1^. Fractions were pooled based on activity and purity, concentrated to ~1 mM for both proteins, and exchanged into 10 mM MOPS, pH 7.5, containing 50 mM NaCl. The purified protein was drop frozen in liquid N_2_ and stored at −80 °C.

### Purification of T4moF

T4moF cell paste was re-suspended in 25 mM MOPS, pH 7.5, containing 80 mM NaCl and 2% glycerol at a ratio of 1.5 ml per g of wet cell paste^55^. The cell-free extract was prepared as described above for T4moH. The supernatant was carefully decanted and diluted with two volumes of the above buffer and loaded onto a DEAE Sepharose column (45 mm diameter × 250 mm, GE Health Care) equilibrated in the above buffer and eluted in an 80–400 mM NaCl gradient in the same buffer at a flow rate of 5 ml min^−1^. T4moF was pooled based on the colour of the flavin adenine dinucleotide and [2Fe-2S] cofactors and catalytic activity. Purity was monitored by SDS–PAGE. Pooled fractions were concentrated and applied to a Sephacryl S-100 (45 mm diameter × 1,000 mm, GE Health Care) column equilibrated with 25 mM MOPS, pH 7.5, 200 mM NaCl and 5% glycerol at a flow rate of 3.33 ml min^−1^. Fractions were pooled based on activity and purity, concentrated to ~1 mM and exchanged into 10 mM MOPS, pH 7.5, containing 50 mM NaCl. The purified protein was drop frozen in liquid N_2_ and stored at −80 °C.

### Steady-state catalysis

Reactions with toluene were performed in 10 ml crimp-sealed vials in 50 mM sodium phosphate, pH 7.5, containing 50 mM NaCl, 3 mM NADH, saturating substrate, 4 μM T4moH, 0.4 μM T4moF and varying amounts of T4moC and T4moD. The reactions were quenched with NaCl-saturated 2 N HCl, and extracted with chloroform containing 3-methyl benzylalcohol as an internal standard for toluene reactions. Products were quantified by gas chromatography using flame ionization detection (GD-FID)^22^.

### Crystallization and structure determination

Crystals used to solve 4P1C were obtained by mixing 1.5 μl of T4moH (100 μM) and 1.5 μl of T4moC (200 μM) with an equal volume of 0.1 M MOPS/HEPES, pH 7.5, containing 20% PEG 3350, 200 mM ammonium chloride, 5% Jeffamine (Hampton Research) and 10 mM MgCl_2_. After ~1 week at 19 °C, large thin petal-like crystals formed with dimensions (μm) of ~200 × 500 × 25. Crystals used to solve 4P1B were obtained from hanging drop vapour diffusion by mixing 1.5 μl of a mixture of T4moH (100 μM) and T4moC (200 μM) with an equal volume of 0.1 M Bis-Tris, pH 6.0, containing 18% PEG 3350, 200 mM ammonium acetate, 5% Jeffamine and 10 mM MgCl_2_. After ~1 week at 19 °C, clusters of crystals were formed. Crystals of 4P1C and 4P1B were cryo-protected in Fomblin 2500 and frozen in liquid N_2_. Diffraction data were collected at the Life Sciences Collaborative Access Team (LS-CAT) at the Advanced Photon Source (Argonne National Lab). Diffraction data for 4P1C were collected at station 21-ID-G *λ*=0.97857 Å, whereas data for 4P1B were collected at station 21-ID-D *λ*=0.97857 Å, both at 100 K. The data were indexed, integrated and scaled using HKL2000 (ref. 56). Phase solutions were obtained from CCP4 MolRep by using one protomer of T4moH PDB accession 3DHG as the starting model^57^. Electron density was fit and refined in multiple iterations using Phenix.refine^57^ and Coot^58^. Ramachandran and rotamer analysis were performed using MolProbity^59^. Ramachandran analysis (%) for 4P1C resulted in 96.48 favourable, 3.42 allowed and 0.10 disallowed conformations, whereas analysis for 4P1B gave 97.0 favourable, 2.86 allowed and 0.16 disallowed conformations. Protein–protein interface surface areas were calculated using PISA^60^. Predictions of preferred electron transfer pathways were from HARLeM^43^. Figures were prepared using Pymol^61^.

## Author contributions

J.F.A designed experiments, obtained crystals, solved and refined structures, analysed data and wrote the manuscript. L.J.B. designed experiments, obtained initial crystallization results and analysed data. N.L.E. designed exeriments, performed enzymatic assays and analysed data. B.G.F. designed experiments, analysed data and wrote the manuscript. All authors discussed results and commented on the manuscript.

## Additional information

**Accession codes:** Atomic coordinates and structure factors have been deposited in the Protein Databank under the accession codes 4P1B and 4P1C, respectively.

**How to cite this article:** Acheson, J. F. *et al.* Structural basis for biomolecular recognition in overlapping binding sites in a diiron enzyme system. *Nat. Commun.* 5:5009 doi: 10.1038/ncomms6009 (2014).

## Supplementary Material

Supplementary Information Supplementary Figures 1-6, Supplementary Tables 1-2.

## Figures and Tables

**Table 1:** Data collection and refinement statistics.

	**4P1C**	**4P1B**
*Data collection*		
Space group	P2_1_2_1_2_1_	P3_2_21
		
*Cell dimensions*		
*a*, *b*, *c* (Å)	95.22, 106.35, 213.42	128.37, 128.37, 284.46
α, β, γ (°)	90, 90, 90	90, 90, 120
Resolution (Å)	47.69–2.40 (2.43–2.40)*	42.56–2.05 (2.07–2.05)
*R*_sym_	0.129 (0.76)	0.118 (0.65)
*I*/σ*I*	12.4 (2.0)	6.6 (2.2)
Completeness (%)	95.10 (93.00)	100 (100)
Redundancy	6.0 (4.5)	8.4 (7.6)
		
*Refinement*		
Resolution (Å)	47.69–2.40	42.56–2.06
No. of reflections	81,284/6649	188,039/12,518
*R*_work/_*R*_free_	0.153/0.215	0.146/0.177
		
*No. of atoms*		
Protein	16,137	16,269
Ligand/ion	24	66
Water	609	1,917
		
*B-factors*		
Protein	20.17	28.87
Ligand/ion	21.36	39.71
Water	19.92	38.08
		
*R.m.s deviations*		
Bond lengths (Å)	0.008	0.008
Bond angles (°)	1.077	0.859

r.m.s., root mean square.

Each data set was collected from a single crystal.

^*^Data collection statistics for the highest resolution shell are shown in parenthesis.
