# Supplementary material for: Structural basis for biomolecular recognition in overlapping binding sites in a diiron enzyme system
Source: Nat Commun. 2014 Sep 24;5:5009. doi: 10.1038/ncomms6009 (PMC4200526; doi:10.1038/ncomms6009)
Supplement: Supplementary Information — Supplementary Figures 1-6, Supplementary Tables 1-2. [file ncomms6009-s1.pdf]

Supplementary Information

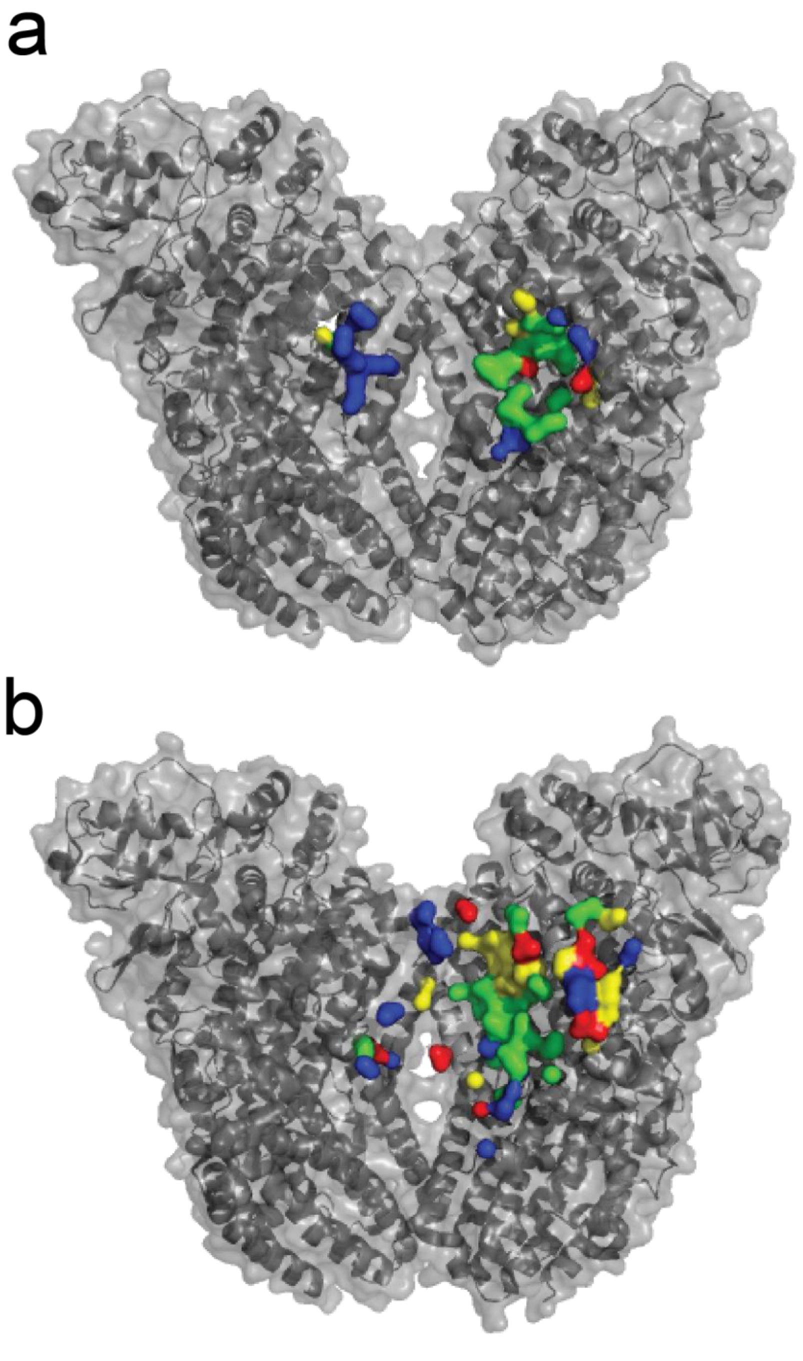

Supplementary Figure 1 | Binding interface residues in T4moH complexes. a, the T4moHC complex utilizes electrostatic (*blue* positive, *red* negative) and polar (green) residues to provide a productive complex. **b**, the T4moHD complex utilizes additional nonpolar interactions (*yellow*) while encompassing the entire T4moC binding area.

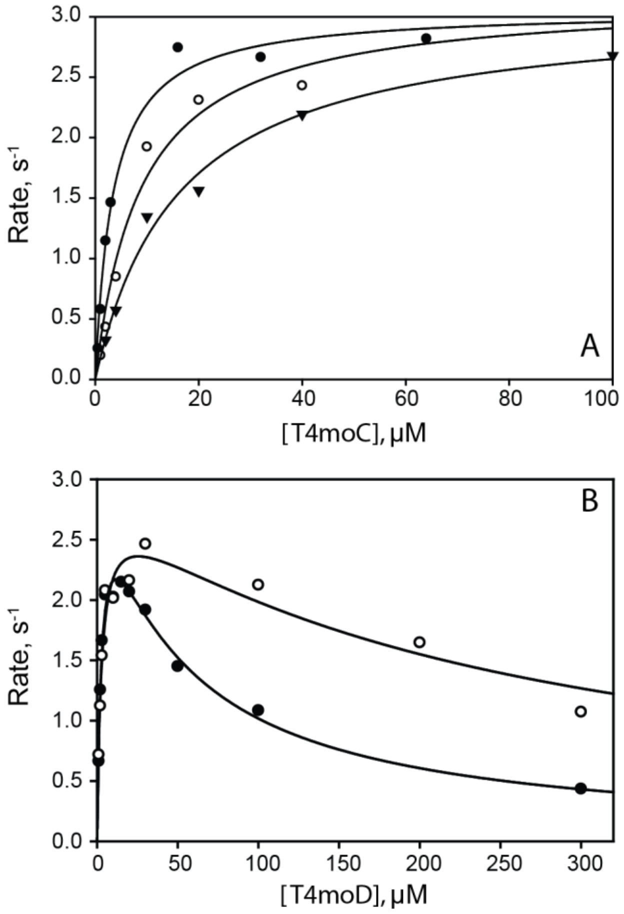

Supplementary Figure 2 | Inhibition of *p*-cresol formation by interplay of T4moC and T4moD binding. Turnover rate is reported relative to the concentration of an (ABE) protomer of T4moH. *A*, solid lines are non-linear least squares fits to $v = k_{cat}\,[\,S\,]/(K_M + [\,S\,])$, with K_M and k_{cat} values for each curve are listed in Table S2. Each curve was constructed by varying T4moC in the presence of a fixed concentration of T4moD: (●) 8μM; (○) 24μM; (▼) 50μM T4moD. *B*, Solid lines are non-linear least squares fits to $v = k_{cat}\,[\,S\,]/(K_M + [\,S\,] + ([\,S\,]^2/K_I))$, with K_M, K_I, and k_{cat} values for each curve are listed in Table S2. Curves were constructed by varying T4moD in the presence of a fixed concentration of T4moC. (●) 12μM T4moC, (○) 42μM T4moC.

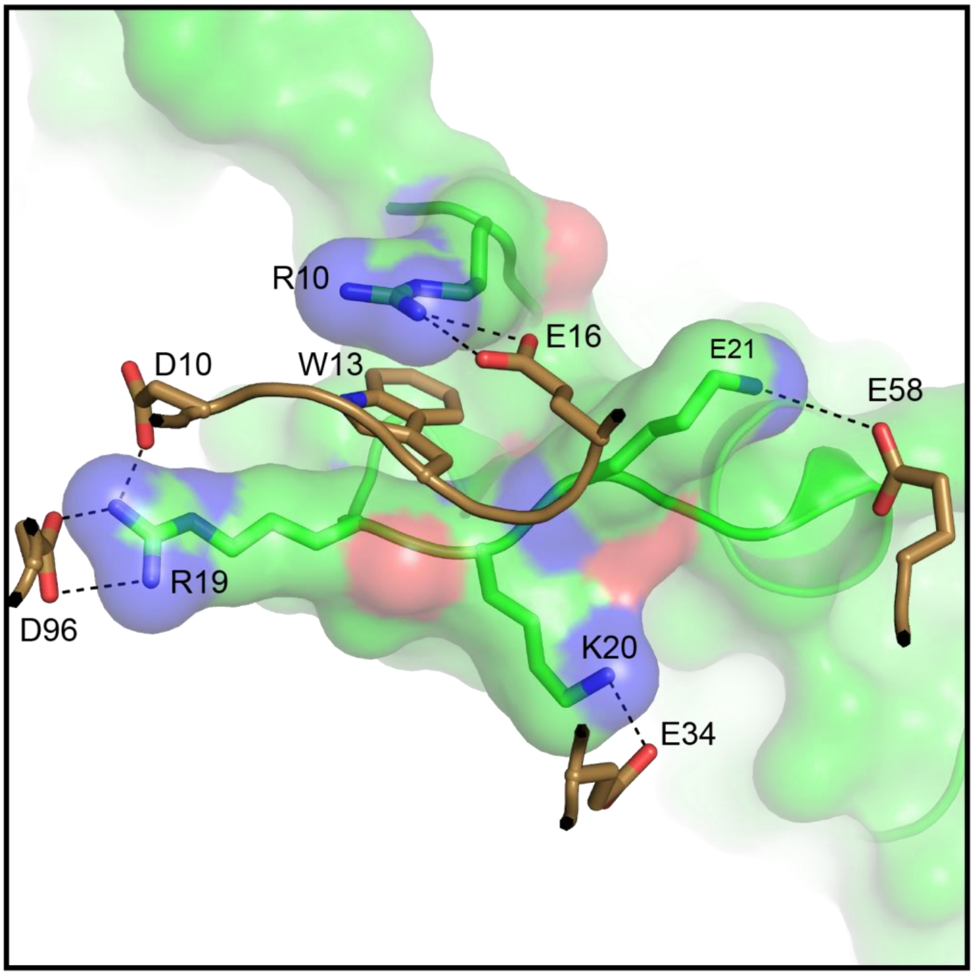

Supplementary Figure 3 | Alternative view of TmoE-T4moC Interface. Binding site formed around T4moC residue W13 (*sand* cartoon and sticks) from TmoE residues E/10-21 (*green* surface and sticks). In addition to the interaction between C/W13 and E/R10, salt-bridges are formed between T4moC/D10 and T4moC/D96 with TmoE/R19, T4moC/E16 and TmoE/R10, T4moC/E34 and TmoE/K20, and T4moC/E58 and TmoE/K21. An orthogonal view of this interface is given in Fig. 3.

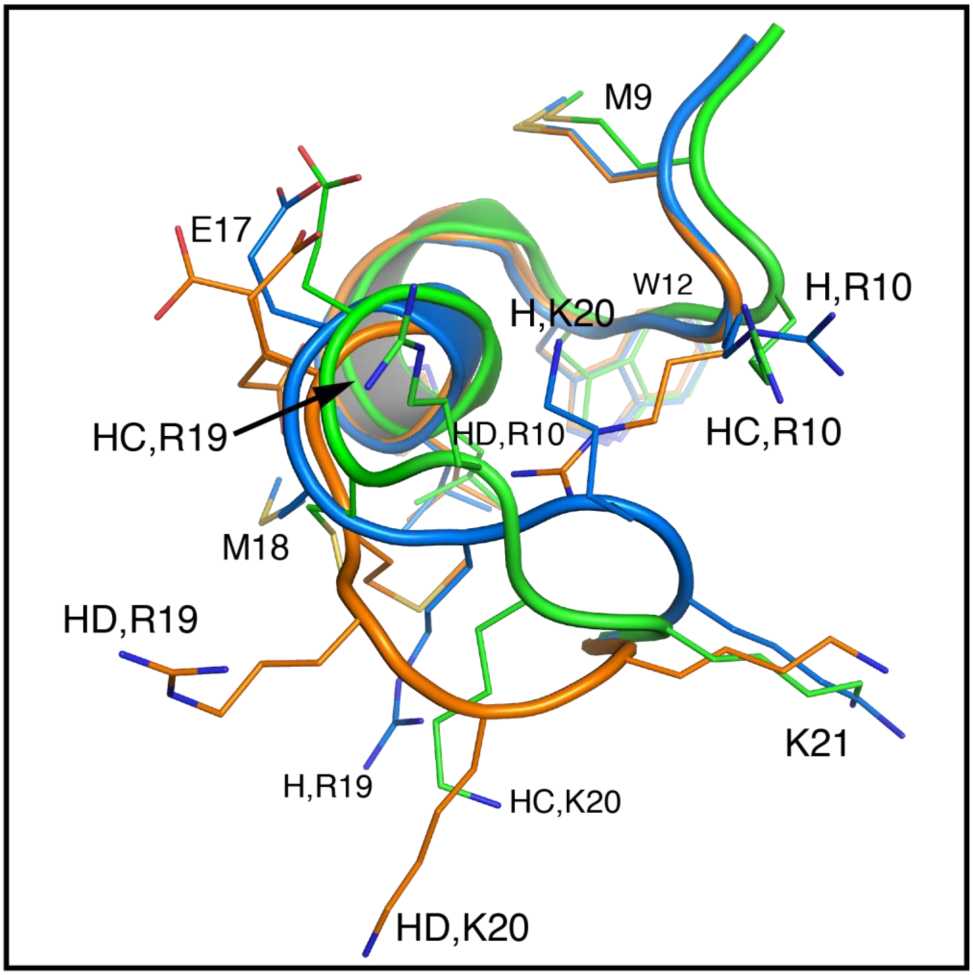

Supplementary Figure 4 | Overlay of TmoE residues 8-22. Residues in this loop adopt different conformations due to crystal packing or protein-partner binding. In T4moH (*marine*), the residues adopts positions based on intereactions with an adjacent dimer in the crystal lattice. When T4moC binds (*green*), they adopt a second configuration to accommodate T4moC W13 (Supplementary Fig. 3). A third orientation is seen as a result of minor interactions with T4moD (*orange*). The most striking differences are for TmoE residues R10, R19, and K20. Each are involved in specific binding contacts with T4moC.

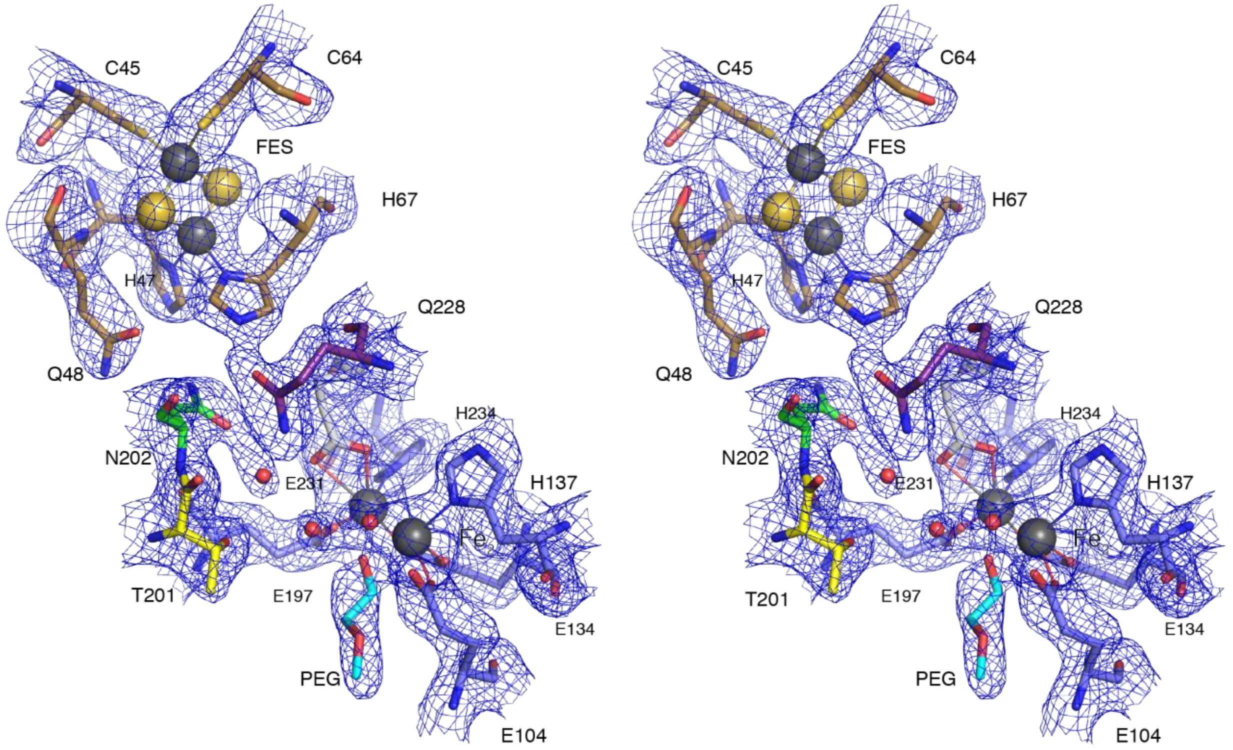

Supplementary Figure 5 | Stereo image of electron transfer region of T4moHC with 2Fo-Fc density maps. Electron density is shown for the residues and cofactors involved in the ET interface (blue 1.0 carve 1.75). The maps were generated in phenix and images were generated by isomesh in pymol. Density for a PEG ligand (*cyan*), a frequently observed feature in T4moH structures, is also shown.

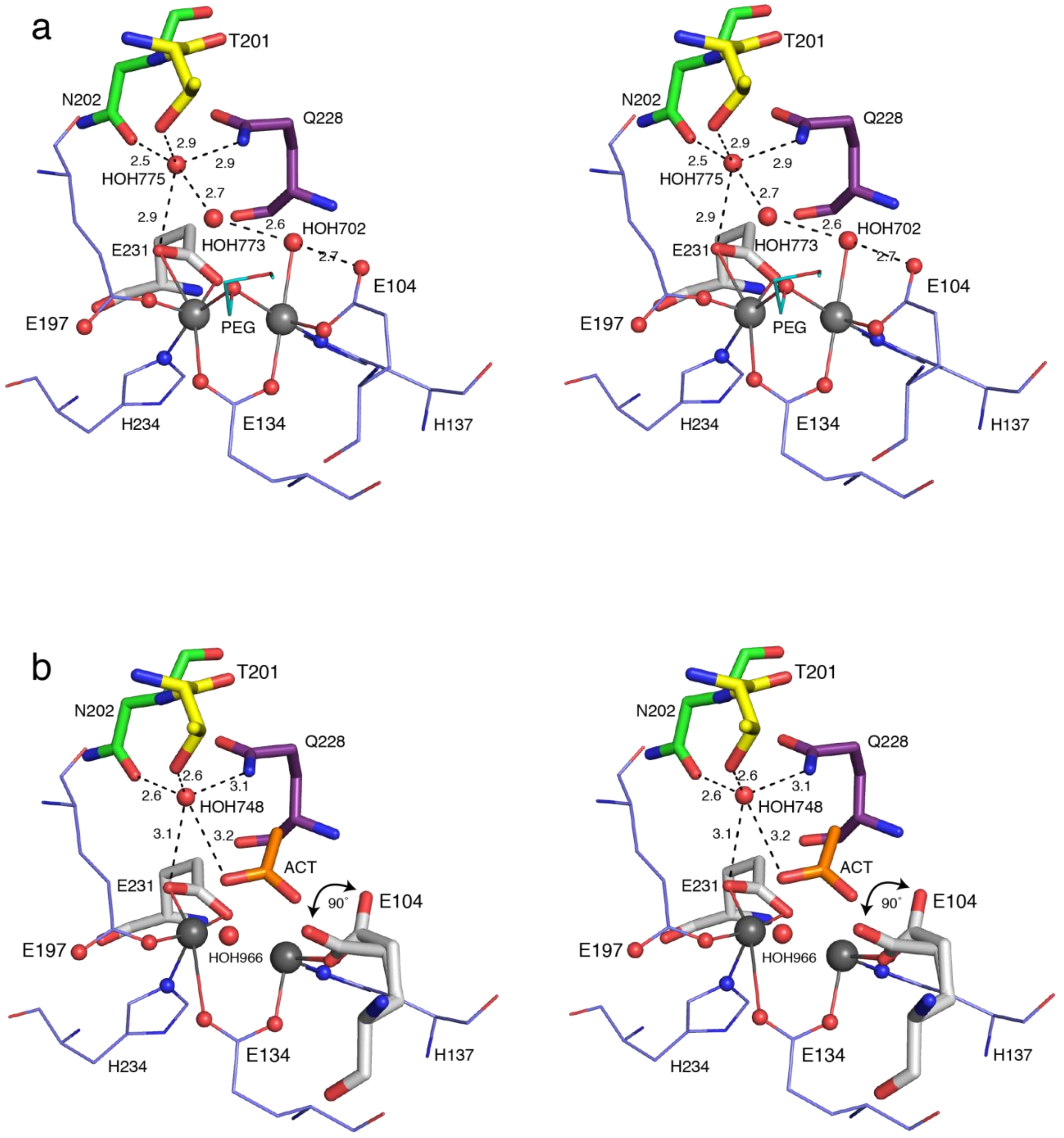

Supplementary Figure 6 | Stereo images of T4moH active sites in the T4moHC complexes. a, active site of 41PC. With the exception of mobile ligand E231 (*grey* sticks), diiron ligands are shown as *blue* lines. E231 has a bidentate coordination to Fe2 that is distinct from monodentate coordination in the diferric states, and μ-carboxylato bidentate coordination in diferrous T4moH and T4moHD. A bridging PEG molecule is shown in *cyan*. N202 and Q228 are also shifted in the 4P1C complex, and a new hydrogen bonded network of water molecules includes HOH775, HOH773 and HOH702. **b**, active site of 41PB. An acetate molecule in a *trans* position relative to bridging E134, while E104 was observed in two monodentate configurations, with hydrogen bonding to either bound acetate (*orange*), or an adjacent active site HOH966.

Supplementary Table 1 | Comparison of dimer, protomer and subunits in T4moHC structures.

Polypeptide chain[1]	RMSD in Cα positions ($Å^2$)
Total $(ABE)_2$	0.451
Protomer (ABEC)	0.176
TmoA subunits	0.161
TMOB subunits	0.150
TMOE subunits	0.126
T4moC	0.187

[1]Except in the case of total $(ABE)_2$ the average of the six possible alignments is reported.

Supplementary Table 2 | Kinetic parameters for reaction of T4MO complexes.

Varied Component	Fixed Component	Ratio T4moD:T4moH[a]	k_{cat} (s^{-1})[b]	Varied component K_M (μM)	Varied component K_I (μM)
	T4moD				
T4moC (Ferredoxin)	8	4	3.1 (0.1)[c]	3.4 (0.5)	
	24	12	3.2 (0.2)	9 (2)	
	50	25	3.1 (0.1)	16 (2)	
	T4moC				
T4moD (Effector)	12	varied	3.3 (0.2)	3.1 (0.5)	45 (7)
	42		2.9 (0.2)	2.7 (0.6)	240 (60)

[a]Reported as a ratio between T4moD and T4moH dimer. [b]Apparent k_{cat} values reported with respect to an (ABE) protomer of T4moH. [c]Error values in parentheses are one standard deviation.
